# Failure and Energy Evolution Characteristics of Saturated Natural Defective Material Under Different Confining Pressures

**DOI:** 10.3390/ma18092027

**Published:** 2025-04-29

**Authors:** Zhihao Gao, Shihao Guo, Xiaoyong Yang, Shanchao Hu, Junhong Huang, Yafei Cheng, Dawang Yin, Jinhao Dou

**Affiliations:** 1 College of Energy and Mining Engineering, Shandong University of Science and Technology, Qingdao 266590, China; 19862869727@163.com (Z.G.); skd994527@sdust.edu.cn (S.H.); qaz1253466914@126.com (J.H.); 15726495680@163.com (Y.C.); m15285817719@163.com (D.Y.); djh15092318906@163.com (J.D.); 2 Shandong Energy Group Xibei Mining Company Limited, Xi’an 710018, China; hjmskd2021@163.com

**Keywords:** natural defective brittle materials, saturated experiment, confinement effect, damage characteristics, energy evolution

## Abstract

In nature, many brittle materials contain natural defects such as microcracks or joints, for example, rocks. Under water-saturated conditions, the strength of defective materials undergoes varying degrees of attenuation, leading to material failure and even structural instability in engineering contexts. Moreover, the deformation and failure of defective brittle materials are essentially the result of the accumulation and dissipation of energy. Studying the energy evolution of defective brittle materials under load is more conducive to reflecting the intrinsic characteristics of strength changes and overall failure of brittle materials under external loading. Natural defective brittle rock materials were firstly water saturated and triaxial compression tests were performed to determine the mechanical properties of water-saturated materials. The energy evolution patterns of water-saturated materials under varying confining pressures were also obtained. Using the discrete element method, the macro- and micro-failure characteristics of water-saturated materials were investigated, revealing the mesoscopic mechanisms of deformation and failure evolution in these materials. The results indicate that confining pressure significantly enhances the peak compressive strength and elastic modulus of water-saturated defective materials. When the confining pressure increased from 0 MPa to 20 MPa, the peak strength and elastic modulus of the water-saturated materials increased by 126.8% and 91.9%, respectively. Confining pressure restricts the radial deformation of water-saturated materials and dominates the failure mode. As confining pressure increases, the failure mode transitions from tensile splitting (at 0 MPa confining pressure) to shear failure (at confining pressures ≥ 10 MPa), with the failure plane angle gradually decreasing as confining pressure rises. Confining pressure significantly alters the energy storage–release mechanism of water-saturated defective brittle materials. At peak load, the total energy, elastic energy, and dissipated energy increased by 347%, 321%, and 1028%, respectively. The ratio of elastic energy storage to peak strain ratio shows a positive correlation, and the elastic storage ratio of water-saturated defective brittle materials under confining pressure is always higher than that without confining pressure. When the strain ratio exceeds 0.94, a negative correlation between confining pressure and the rate of elastic storage ratio is observed. From the perspective of mesoscopic fracture evolution in water-saturated defective brittle materials, the crack propagation path shifts from the periphery to the center of the material, and the fracture angle decreases linearly from 89° to 58° as confining pressure increases. The dominant direction of crack development is concentrated within the 45–135° range. The findings elucidate the mechanisms by which water saturation and confining pressure influence the strength degradation of natural defective brittle materials from both mesoscopic and energy perspectives, providing theoretical support for the stability control of related engineering structures.

## 1. Introduction

Naturally defective materials, as the most common engineering media in underground construction [1,2,3], undergo significant changes in mechanical properties due to the combined effects of in situ stress and groundwater as underground projects extend to greater depths [4,5,6]. These changes often lead to engineering hazards, making it crucial to understand the deformation-failure characteristics and energy evolution mechanisms of water-saturated defective materials under varying confining pressures to ensure the stability of deep underground structures [7,8].

In recent years, extensive research has been conducted on the mechanical behavior of naturally defective materials under different moisture conditions. Colback et al. [9] investigated the mechanical properties of quartz sandstone with varying moisture contents, revealing that the strength of saturated quartz sandstone is approximately 50% of that in its dry state. Li et al. [10] demonstrated through mechanical tests that specimens with higher initial strength exhibit greater compressive resistance under the same initial density and moisture content. Song et al. [11] conducted uniaxial compression tests on fractured sandstone in both natural and hydrochemical environments, finding that water significantly influences crack initiation and failure modes. Chen et al. [12] observed that water accelerates crack propagation in shale under varying moisture conditions. Zhao Yanlin et al. [13] demonstrated through permeability tests that pore water pressure negatively correlates with rock strength and elastic modulus. Existing studies confirm that rock failure is fundamentally driven by energy release [14,15,16,17,18]. Xie Heping et al. [19] proposed that rock damage results from the interplay between energy accumulation and dissipation during deformation. Meng Zhaoping et al. [20] investigated the influence of moisture content on elastic strain energy in sedimentary rocks under uniaxial compression. Lai Xingping et al. [21] explored the patterns of energy release in coal-rock masses with varying moisture contents. Liu Wanli et al. [22] analyzed the energy transformation characteristics of saturated mudstone through uniaxial compression tests. Liu Yunsi et al. [23] conducted Brazilian splitting tests on saturated slate using an SHPB system, showing that the dissipated energy density of saturated slate exceeds that of dry slate at higher loading rates. Guo Jiaqi et al. [24] revealed that saturated karst limestone exhibits faster energy release rates compared to dry samples. Gao et al. [25] established a damage evolution model for freeze–thaw cycled sandstone based on energy dissipation ratios. Yang Yongjie et al. [26] developed a damage variable model for coal samples by studying energy evolution under different loading-unloading paths. Zhao Yixin et al. [27] examined the effects of moisture on energy density and damage variables in coal through Brazilian splitting tests. Huang Ming et al. [28] investigated the energy dissipation characteristics of water-bearing siltstone under impact loading. Peng et al. [29] found that the energy absorption efficiency of CASS materials decreases with increasing strain rates. Jiang Jingdong et al. [30] quantified the relationships between total energy, elastic stored energy, and moisture content in triaxial compression tests.

Although previous research has emphasized the significant influence of moisture content on the mechanical properties and energy damage characteristics of rocks, studies addressing the combined effects of confining pressure and water saturation on the failure behavior and energy evolution of naturally defective materials remain limited. To bridge this gap, the present study conducts uniaxial and triaxial compression experiments on dry and saturated defective materials under varying confining pressures. Specifically, the study systematically examines the following: (i) the variation in mechanical parameters under different confining pressures and saturation conditions; (ii) the macro-failure patterns and meso-damage evolution mechanisms of saturated defective materials subjected to varying confining pressures; and (iii) the mechanisms of energy accumulation and dissipation in water-saturated materials under confining pressure gradients. This research aims to provide theoretical foundations for the stability assessment and disaster mitigation of deep underground engineering projects.

## 2. Water-Saturated Test Material Preparation and Programs

### 2.1. Water-Saturated Defective Brittle Material Specimen Preparation

**Figure 1:**
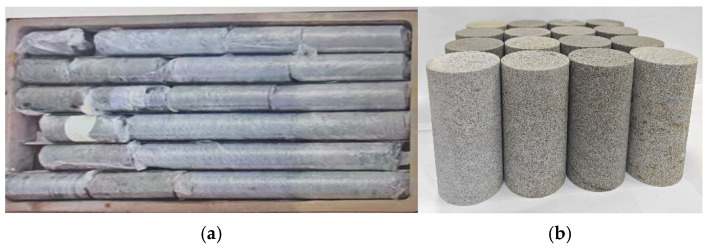
Natural defective brittle material specimen: (**a**) engineering drill core; (**b**) indoor test samples.

The natural defective brittle material used in the test was sourced from the western Ordos mining area in Inner Mongolia, extracted from middle sandstone at a burial depth of approximately 600 m. The samples exhibited no obvious surface cracks and were gray in their natural state. The natural defective brittle material specimens are shown in Figure 1. The surface of the material was rough, and the specimens were cylindrical with a diameter of 89 mm and a column length between 10 and 70 cm. The engineering drilling core is shown in Figure 1a. According to the “engineering rock test method standard”, the retrieved core was subjected to secondary processing, including cutting, polishing, and shaping into standard cylindrical specimens with a height of 100 mm and a diameter of 50 mm, as shown in Figure 1b. The height error of the standard specimen was controlled within 2 mm, and the unevenness of the two end surfaces was maintained within 0.05 mm.

**Figure 2:**
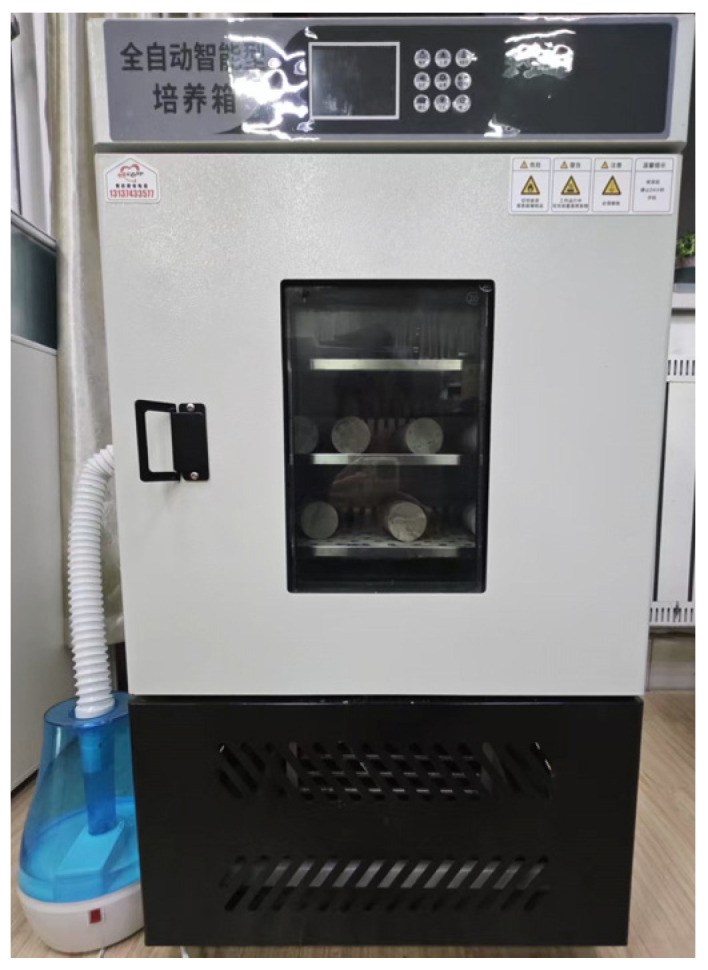
Constant temperature and humidity maintenance box.

First, the processed specimens were placed into the drying box shown in Figure 2. The temperature of the drying box was set to 110 °C, and the specimens were weighed every 6 h. If the difference in mass between two consecutive weighings was no more than 0.1 g, the specimens were considered to have reached a completely dry state. The water-saturated specimens were prepared in accordance with the Standard for Test Methods of Engineering Rock Masses. The procedure involved rapid water absorption of dried samples using a free immersion method, followed by 48 h immersion. Subsequently, a vacuum saturation process was implemented for 6 h to ensure complete bubble elimination. After a 4 h stabilization period, the specimens were weighed to confirm full saturation.

### 2.2. Characterization of the Basic Mineral Composition and Microstructure of Natural Defective Materials

**Figure 3:**
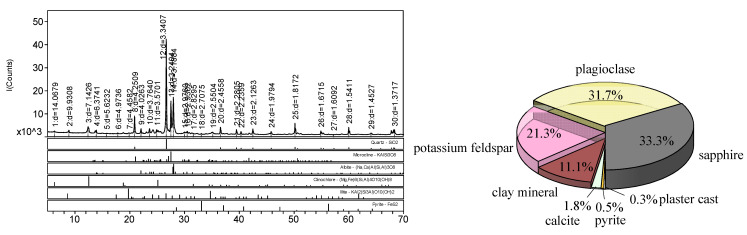
XRD diffraction and mineralogical composition content map.

To analyze the mineral composition of defective rock brittle material, powder sample from the core retrieved at the site was manually ground until the particle size was smaller than a 325-mesh sieve. The powder sample was then evenly spread on a glass sample tank to form a specimen, which was placed into the test instrument for X-ray diffraction analysis. By utilizing the different lattice spacings between the mineral components and comparing them with the corresponding reference cards and diagrams, the mineral composition and content of the specimen were determined. As illustrated in Figure 3, the experimental results reveal that the medium sandstone specimen contains seven mineral compositions: quartz, plagioclase feldspar, potassium feldspar, clay minerals, calcite, pyrite, and gypsum. The primary component of clay minerals is chlorite.

**Figure 4:**
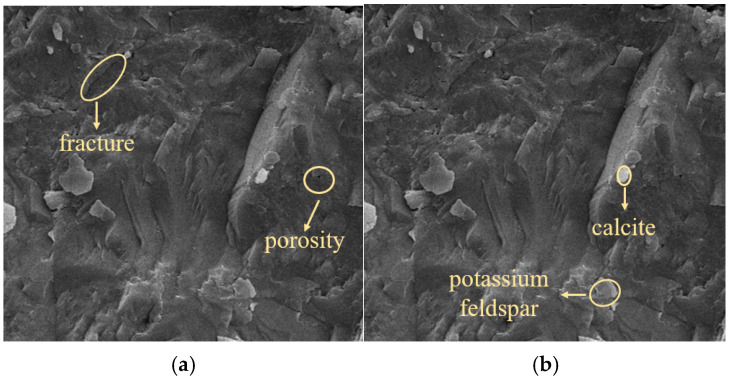
Microstructure of defective sandstone materials: (**a**) fine structure of defective sandstone materials; (**b**) defective sandstone material components.

Based on the complex composition of the mineral components in the natural defective rock brittle materials, and combined with the micro-morphological structure tests, the analysis was conducted using thermal field emission scanning electron microscopy. The micro-morphological structure of the materials is shown in Figure 4. Figure 4a shows that the surface of the defective rock material is flat, with particles tightly bound to each other, although a small number of cracks and pores are still present. Figure 4b reveals the presence of clay minerals such as calcite, potassium feldspar, etc., which react and expand in contact with water, making the pore cracks in the rock material to further expand.

### 2.3. Test Setup and Test Program

**Figure 5:**
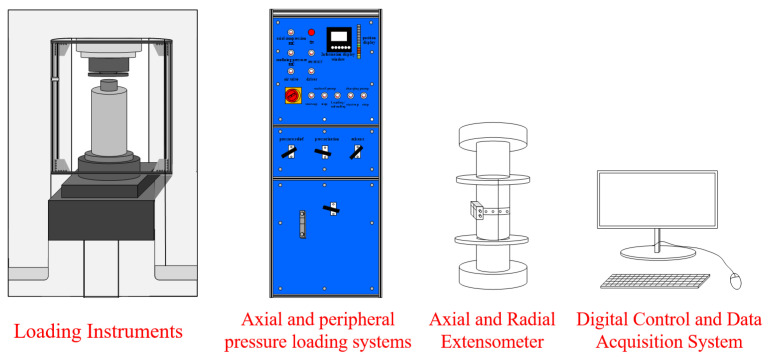
ZST rock triaxial test system.

The buried depth of the defective brittle material used in the test is about 600 m, with the confining pressure around 13.8 MPa. For the test, a confining pressure of 10 MPa is selected as the initial value. The test loading instrument adopts the ZST series rock triaxial system, which includes an axial loading system, a confining pressure loading system, and a digital control and data acquisition system. The axial loading system, along with the radial deformation-induced gauge, can employ either displacement or force loading methods. The confining pressure loading system utilizes a hydraulic oil pump for loading, with the loading process and data collection controlled by a computer control system. The test loading device is shown in Figure 5 below. The force loading method is adopted, with a loading accuracy of 1%, and the loading termination condition is set as the destruction of the specimen. To study the effect of confining pressure on the mechanical properties and energy evolution of the materials in a water-saturated state, the confining pressures were set to 0, 10, 15, and 20 MPa, resulting in four different confining pressure conditions. The specific test program is shown in Table 1. To ensure low test dispersion, three groups of tests under the same confining pressure conditions were conducted as controls.

## 3. Mechanical Properties and Deterioration Law of Water-Saturated Natural Defective Materials

### 3.1. Stress–Strain Analysis

**Figure 6:**
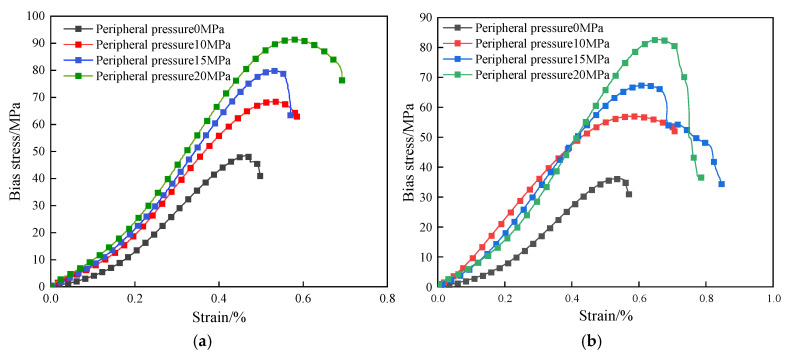
Bias stress–strain curve: (**a**) drying of natural defective specimens; (**b**) water-saturated natural defect specimens.

**Figure 7:**
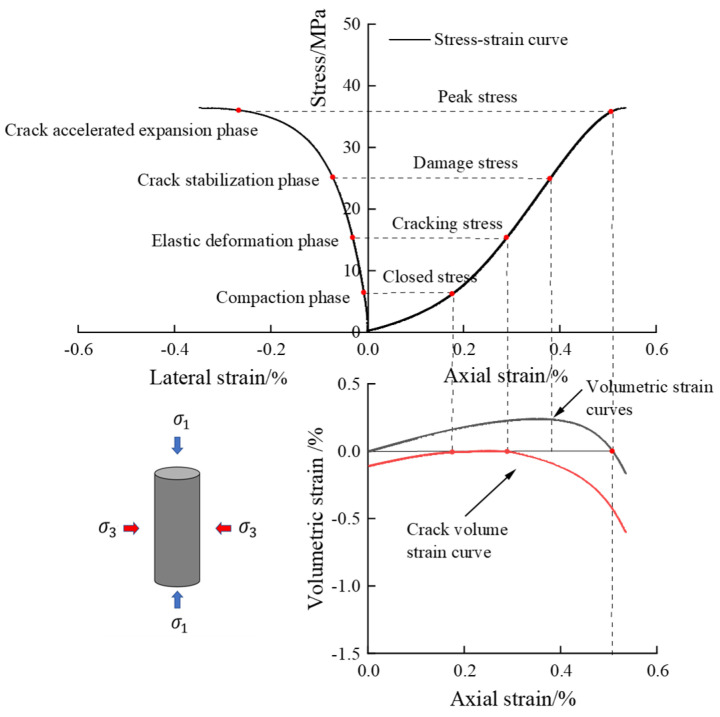
Stress–strain diagram of the damage process of natural defective materials (water-saturated uniaxial).

Based on the analysis of the test results in Figure 6 and Figure 7, the bias stress–strain curves of both dry defective brittle material specimens and water-saturated material specimens under different confining pressure conditions exhibit the same trend. These curves can be divided into four typical deformation phases: the compaction phase, the elastic deformation phase, the crack stabilization phase, and the crack accelerated expansion phase. Under the combined effect of confining pressure and axial pressure, the natural defective specimen first experiences the compaction stage. During this stage, the original pores and microcracks are gradually closed under the action of stress, and the curve displays nonlinear characteristics. As the axial load continues to increase, the curve enters the elastic deformation stage, where no new cracks are formed internally, and the stress and strain exhibit a linear relationship. When the axial load reaches the yield strength, the specimen enters the stable crack development stage, during which new cracks start to develop internally. As the axial load continues to increase towards the yield strength, the specimen transitions into the accelerated crack expansion stage. At this point, cracks penetrate through the material, and main cracks form on the surface. The load-carrying capacity decreases rapidly, and the curve shows decay characteristics.

### 3.2. Analysis of the Law of Change of Mechanical Parameters of Natural Defective Materials

**Figure 8:**
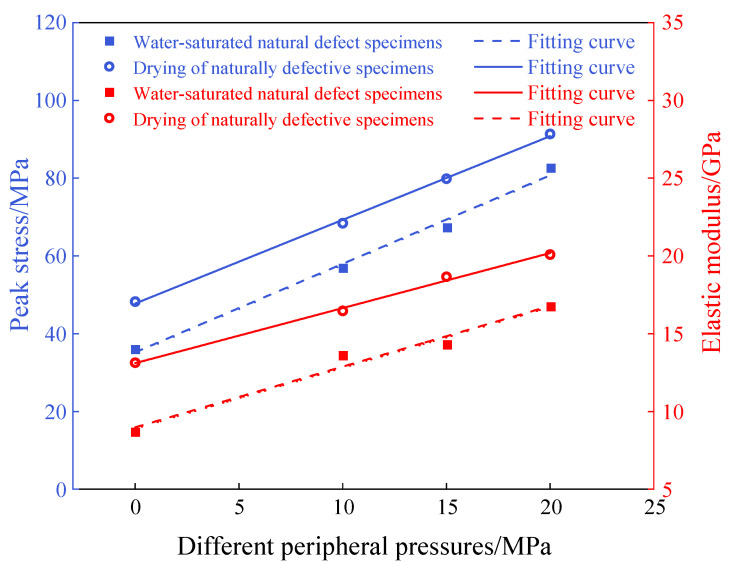
Variation in mechanical parameters of defective brittle materials with increasing confining pressure.

The evolution pattern of conventional triaxial mechanical parameters of defective brittle material under different confining pressures is shown in Figure 8. As the confining pressure increases from 0 MPa to 20 MPa, the peak strength of water-saturated materials increases from 36.18 MPa to 82.17 MPa, a 126.8% increase; the elastic modulus improves from 8.7 GPa to 16.7 GPa, a 91.9% increase. In contrast, the peak strength of dry materials increases from 48.23 MPa to 91.39 MPa, an 89.34% increase, and the elastic modulus increases from 13.13 GPa to 20.09 GPa, a 52.55% increase. Linear regression analysis based on the test data shows that the peak strength and modulus of elasticity of the material under water-saturated conditions are lower than those under dry conditions. The increase in the level of confining pressure has a significant effect on the mechanical parameter of the defective brittle rock material under water-saturated conditions. The confining pressure compacts the cracks within the material, effectively increasing the density of the rock. Additionally, the confining pressure restricts the radial deformation of the material, reducing the tendency for volumetric expansion, and strengthens the normal stresses between rock particles, which improves the particle density. The increase in normal stress between the material particles enhances friction between the particles, further contributing to the overall strength of the material.

**Figure 9:**
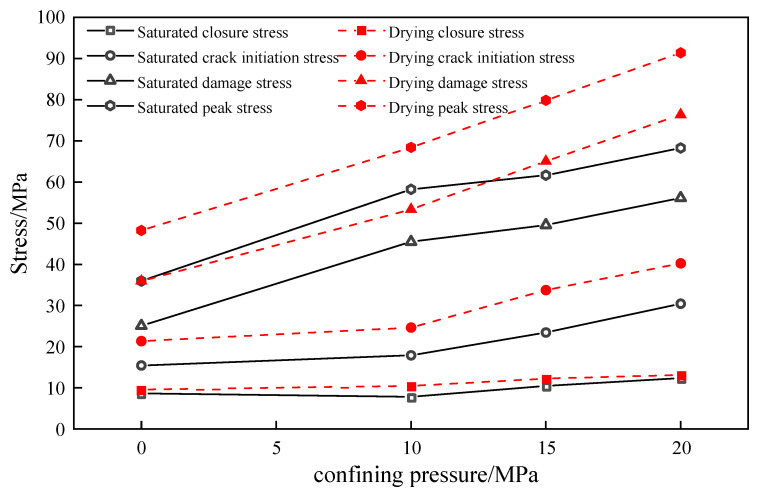
Variation in critical stress with confining pressure for natural defective materials.

The evolution of critical stresses in natural defective brittle materials under different enclosing pressures is shown in Figure 9. The defective rock materials in both dry and water-saturated states exhibited the same growth trend under varying enclosure pressures. However, the critical stresses of dry materials were higher than those of water-saturated materials due to changes in the microstructure of the materials caused by water immersion. When the confining pressure of the water-saturated material was increased from 0 MPa to 20 MPa, the closure stress increased from 8.65 MPa to 12.34 MPa, an increase of 42.7%; the crack initiation stress increased from 15.42 MPa to 30.42 MPa, an increase of 98.7%; and the damage stress increased from 25.07 MPa to 56.21 MPa, an increase of 124.2%. The increase in confining pressure level showed a similar trend for both dry and water-saturated defective rock materials, but the critical stresses of dry material were higher than those of water-saturated materials due to change in the material’s microstructure under the effect of water immersion. The increase in pressure levels strengthens the critical stresses in the water-saturated material in two ways. On the one hand, the increase in confining pressure enhances the internal density of the material, which reduces the extensibility of primary cracks. On the other hand, it enhances the normal force at the particle contact surfaces and strengthens the anti-slip ability of the particles, thereby improving the strength required for the initiation of nascent cracks. The four key stresses—crack closure stress, crack initiation stress, damage stress, and peak stress—represent the complete process of progressive damage in rock materials and exhibit a significant positive correlation with the level of surrounding pressure.

### 3.3. Damage Characteristics of Water-Saturated Natural Defective Rock Materials Under Different Confining Pressures

**Figure 10:**
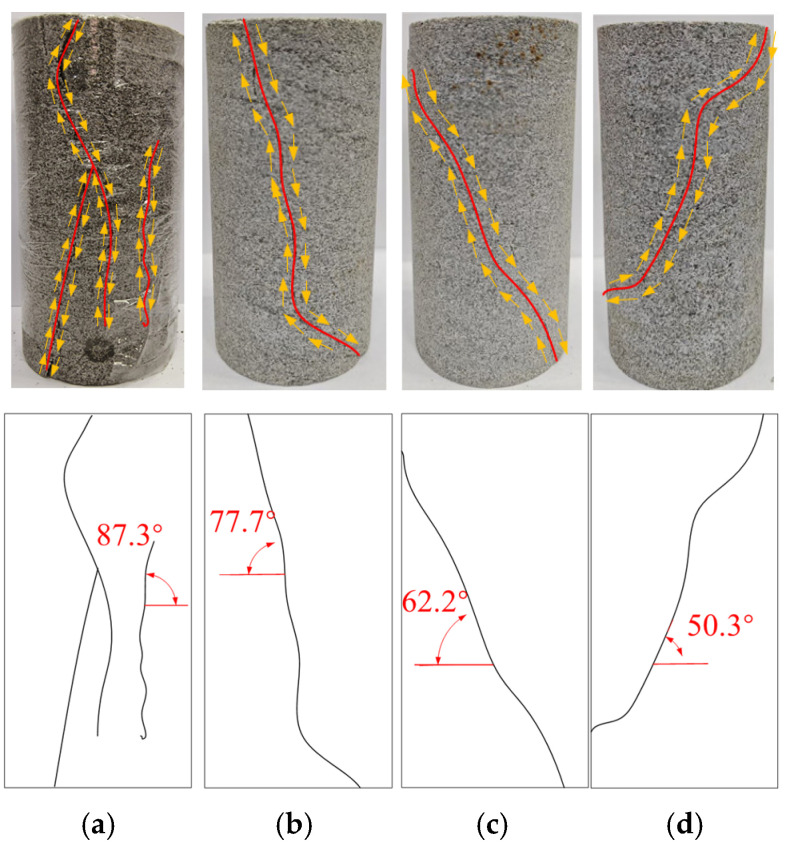
Damage pattern of specimen under different confining pressures: (**a**) confining pressure 0 MPa; (**b**) confining pressure 10 MPa; (**c**) confining pressure 15 MPa; (**d**) confining pressure 20 MPa.

Figure 10 shows the damage characteristics of the water-saturated material under different confining pressure conditions in the triaxial compression test. It is observed that the damage modes of the water-saturated defective rock materials under different confining pressures primarily manifest in two modes: tensile damage (the main cracks are nearly vertical to the horizontal direction) and shear damage (the angle between the main cracks and the horizontal direction is acute). When the confining pressure is 0 MPa, the specimen shows typical longitudinal tensile damage, with the inclination angle of the main cracks around 89°. When the confining pressure increases to 10 MPa, the damage mode transitions to single-slope shear damage, with the main crack inclination angle approximately 77.7°; at 15 MPa, the damage mode remains shear damage, with the main crack inclination angle around 62.2°; and when the confining pressure increases to 20 MPa, the damage mode continues to be shear damage, with the main crack inclination angle around 50.3°. The confining pressure dominates the crack extension path and damage characteristics. In the absence of confining pressure, the material is not affected by the annular constraint force, and stress concentration due to the maximum principal stress direction forms radial tensile stress, leading to a damage mode dominated by tensile damage. However, with the application of confining pressure, the annular constraint force enhances the friction force between the particles, causing the crack extension direction to deviate from the direction of the maximum principal stress. This shift results in a damage mode dominated by shear damage. Therefore, there is a negative correlation between confining pressure and crack inclination angle: as confining pressure increases, the crack inclination angle decreases.

## 4. Numerical Simulation Based on Discrete Element Method

### 4.1. Principle and Parameter Calibration

**Figure 11:**
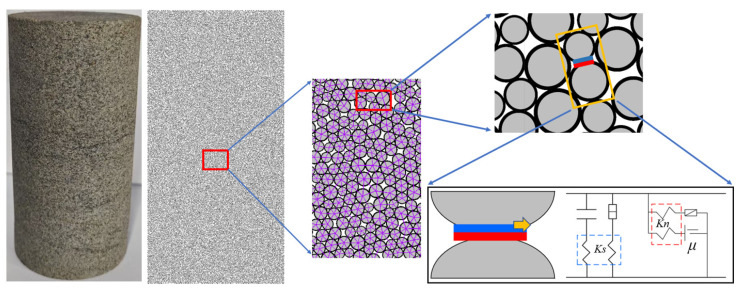
Numerical specimen.

The core of the discrete element method is to map the microscopic mechanical behavior of the material through the mechanical behavior of the particles. This approach allows for the visual demonstration of the dynamic processes of crack generation, expansion and energy conversion, enabling cross-scale mechanical analysis from micro to macro levels. In the indoor experiments, water-saturated material with a density of about 2250 kg·m^−3^ was used, with cylindrical specimens having a diameter of 50 mm and a height of 100 mm. In PFC2D, the specimens were generated with the same dimensions as those in the indoor tests. The minimum particle radius was set at 0.9 mm, with a particle size ratio (maximum radius to minimum radius) of 1.66. The parallel bonding model was applied between the particles, as shown in Figure 11. Based on the loading method of the indoor test, the model was loaded by displacement control, with the loading rate of the upper and lower loading plates set at 0.01 m·s^−1^.

**Figure 12:**
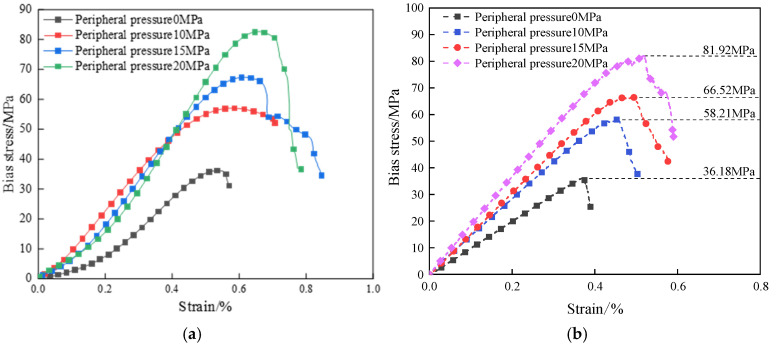
PFC parameter calibration results: (**a**) indoor test curves; (**b**) numerical simulation curves.

To ensure the accuracy of the numerical simulation results, based on the experimental data of uniaxial compressive strength of the specimen, the micro-parameters of the model were systematically calibrated using the trial-and-error method. The destructive characteristics and stress–strain process of the calibration results are shown in Figure 12, and the finalized values of the micro-parameters are shown in detail in Table 2.

### 4.2. Spatio-Temporal Evolution Law of Rift Network

**Figure 13:**
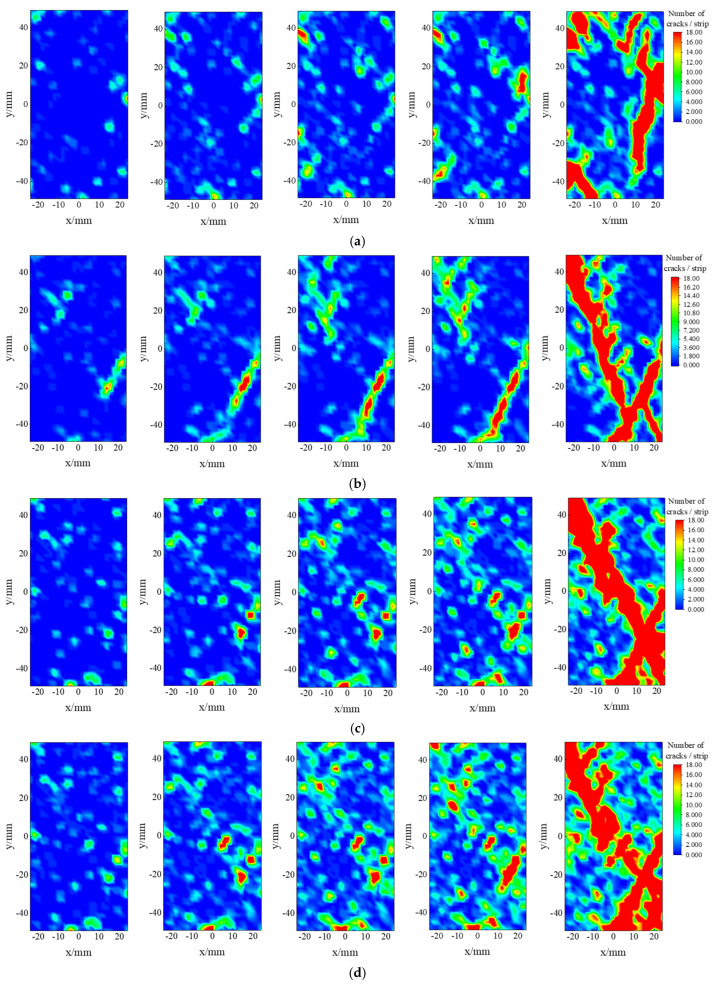
Cracking hotspot map of water-saturated natural defective material under different circumferential pressure conditions: (**a**) confining pressure 0 MPa; (**b**) confining pressure 10 MPa; (**c**) confining pressure 15 MPa; (**d**) confining pressure 20 MPa.

The macroscopic damage nature of defective rock materials under uniaxial and triaxial loading conditions is characterized by the generation, expansion, and penetration of cracks at the microscopic scale. The discrete element method can effectively capture the dynamic development and spatial distribution characteristics of these cracks. As shown in Figure 13, with the increase in bias stress, internal damage in the specimen begins to occur, forming a micro rupture surface with a certain angle to the horizontal direction. The angle between the macro rupture surface and the horizontal plane is the breakage angle. As the confining pressure increases from 0 MPa to 20 MPa, the number of fissures increases, with the fracture angles measured at about 89°, 69°, 63°, and 53°, respectively.

The influence of the surrounding pressure environment on the damage pattern of the defective rock material shows a clear and consistent trend. As the surrounding pressure increases, the crack concentration area (yellow-red area) at the end of the specimen and the boundary area gradually shifts toward the middle of the specimen. Numerical simulation results indicate that, as the confining pressure increases from 0 MPa to 20 MPa, the crack expansion path shifts notably from the free surface to the interior. This suggests that the inhibitory effect of the confining pressure on the internal damage of the material is closely related to the stress redistribution mechanism. Under the influence of confining pressure and stress, the crack tip continues to experience stress concentration, which dominates the direction of crack expansion. Comparative experiments show that, under no confining pressure, the boundary crack hotspot appears early in the axial pressure loading, and the damage area expands rapidly along the free surface. In contrast, under confining pressure, the damage initiation point exhibits multi-point distribution characteristics, and the breakage angle decreases as the confining pressure increases.

### 4.3. Mechanism of Fine-Scale Evolution of Water-Saturated Natural Defective Materials

**Figure 14:**
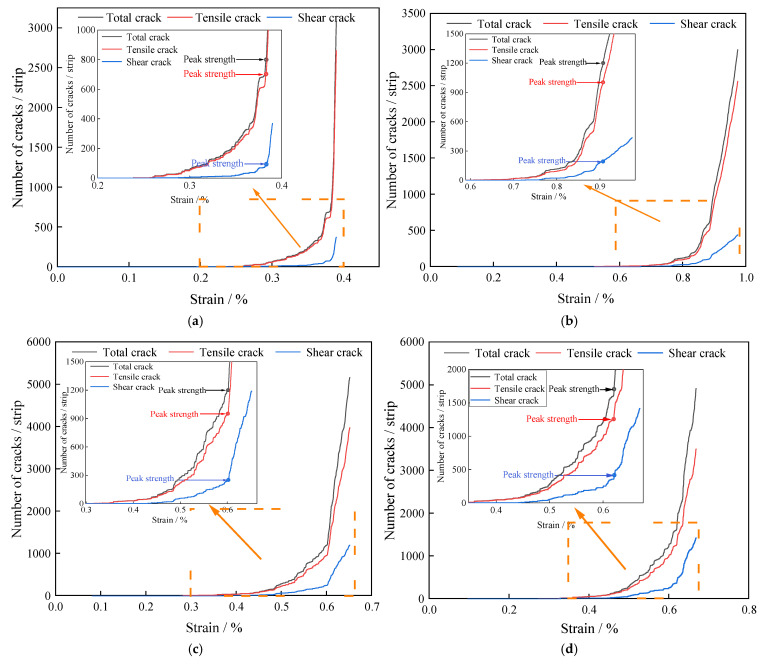
Crack evolution law of water-saturated materials under different confining pressures: (**a**) confining pressure 0 MPa; (**b**) confining pressure 10 MPa; (**c**) confining pressure 15 MPa; (**d**) confining pressure 20 MPa.

Based on the type and number of microcracks generated in the water-saturated material during triaxial loading, as recorded by PFC2D, the evolution of cracks is analyzed under different circumferential pressure conditions. As shown in Figure 14, no cracks are generated inside the water-saturated material during the early stage of loading. As the load increases, tensile cracks are the first to appear, while shear cracks lag behind the tensile cracks before the load reaches the peak value. Therefore, the total cracking trend is similar to that of the tensile cracks. Both shear and tension cracks showed an increasing trend, with the growth rate of tension cracks consistently higher than that of shear cracks. When the confining pressure is 0, 10, 15 and 20 MPa, the ratio of peak strength corresponding to shear cracks to total cracks is 12.1%, 16.3%, 20.8% and 27.7%, respectively. As the confining pressure increases, the ratio of cracks gradually increases, and the peak damage morphology shifts from tension damage to shear damage predominantly. The existence of surrounding pressure has an inhibitory effect on the generation of tension cracks, which in turn leads to a change in the post-peak damage morphology of the water-saturated defective rock material, from tension damage to shear damage.

**Figure 15:**
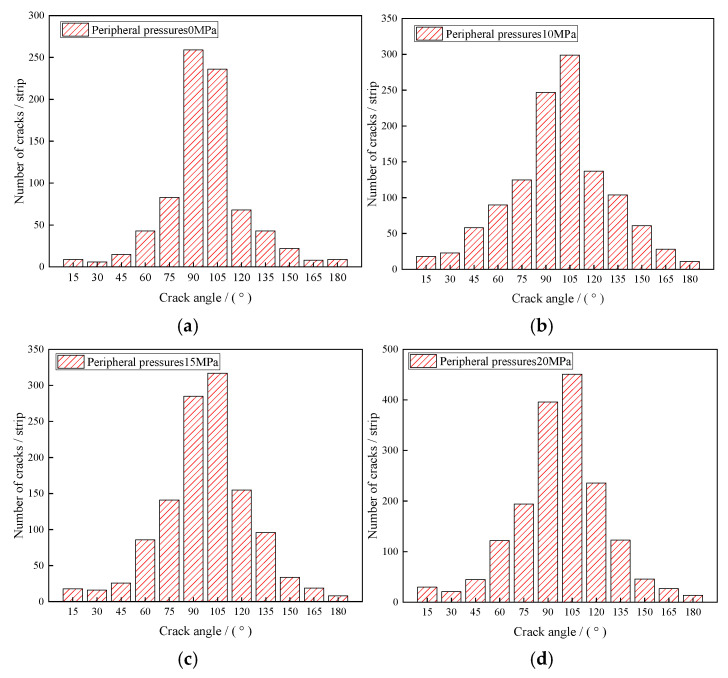
Crack azimuthal distribution under different circumferential pressures: (**a**) confining pressure 0 MPa; (**b**) confining pressure 10 MPa; (**c**) confining pressure 15 MPa; (**d**) confining pressure 20 MPa.

To reveal the evolution mechanism of microcracks in water-saturated materials under triaxial compression, the orientation of cracks in the specimens was counted, as shown in Figure 14, where the horizontal coordinate represents the crack direction and the vertical coordinate represents the number of cracks. As seen in Figure 15, the crack azimuth angle mainly ranges from 60 to 135°, with the highest number of cracks occurring 75–105°. This indicates that, before the peak strength, the peak strength is primarily dominated by internal tension cracks in the water-saturated material. As the circumferential pressure increases, the number of cracks in the 45 to 75° increases, suggesting that the circumferential pressure changes the crack opening angle. This change affects the failure characteristics of the water-saturated material, shifting from vertical damage to inclined damage.

## 5. Mechanisms of Energy Evolution in Water-Saturated Natural Defective Materials

### 5.1. Principles of Energy Calculation

Assuming that the entire test process was a closed system with thermal energy exchange with the external environment, the energy relationship of the defective brittle rock material was derived based on the first law of thermodynamics:

(1) $$U=U_{e}+U_{d}$$

where *U* is the mechanical energy input by the external force during the test; *U_e_* is the elastic energy generated by the elastic deformation; *U_d_* is the dissipated energy generated by the plastic deformation and destruction.

In a conventional triaxial compression test (*σ_2_* = *σ_3_*), the total mechanical energy U of the specimen in this closed system is the sum of the work performed on the specimen by the axial and circumferential pressures, viz:

(2) $$U=U_{1}+U_{3}$$

where *U_1_* is the work performed by axial pressure on the specimen; U_3_ is the work performed by circumferential pressure on the specimen.

The equations for work performed by axial pressure U_1_, work performed by circumferential pressure *U_3_* and elastic strain energy *U_e_* during triaxial compression are given below, respectively [31]:

(3) $$\left.\begin{array}{l} U_{1}=\int \sigma_{1}d\varepsilon_{1}=\frac{1}{2}\sum_{1}^{n}\left(\varepsilon_{1_{i+1}}-\varepsilon_{1_{i}}\right)\left(\sigma_{1_{i}}+\sigma_{1_{i+1}}\right)\\ U_{3}=2\int \sigma_{3}d\varepsilon_{3}=\sum_{1}^{n}\left(\varepsilon_{3_{i+1}}-\varepsilon_{3_{i}}\right)\left(\sigma_{3_{i}}+\sigma_{3_{i+1}}\right)\\ U_{e}=\frac{1}{2E}\left[\left(\sigma_{1}^{2}+2\sigma_{3}^{2}\right)-2\mu \left(2\sigma_{1}\sigma_{3}+\sigma_{3}^{2}\right)\right] \end{array}\right\}$$

where E is the modulus of elasticity in the linear elastic phase during triaxial compression; *μ* is the Poisson’s ratio in the linear elastic phase during triaxial compression.

### 5.2. Characterization of Energy Evolution

**Figure 16:**
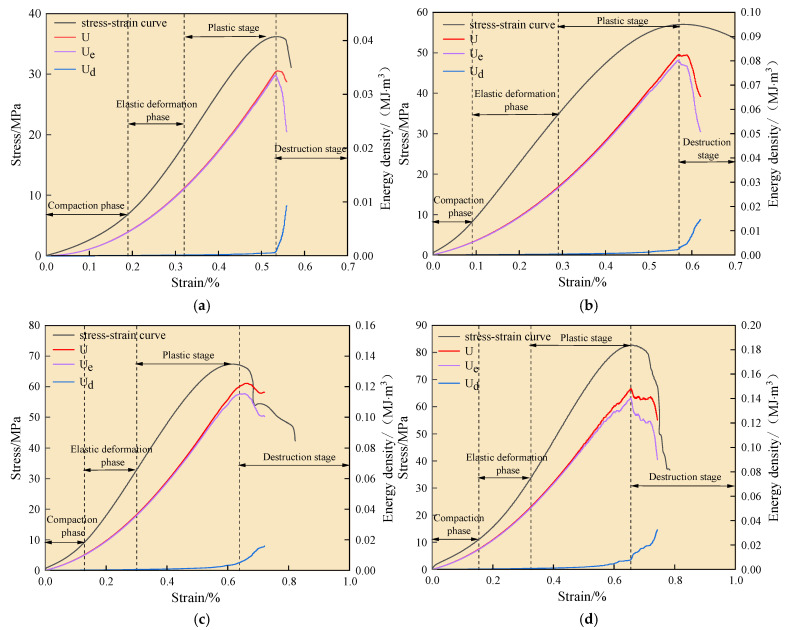
Triaxial compression energy evolution characteristics: (**a**) confining pressure 0 MPa; (**b**) confining pressure 10 MPa; (**c**) confining pressure 15 MPa; (**d**) confining pressure 20 MPa.

The deformation process of water-saturated materials was accompanied by the accumulation and dissipation of energy, and the nature of their destruction was driven by an energy-driven destabilization phenomenon. Therefore, the use of the energy analysis method to study the deformation and damage process of water-saturated materials had significant practical significance. Based on the energy calculation formula, the energy curve was derived, as shown in Figure 16. According to the stress–strain curve, the energy curve can be divided into four stages. In the compression stage, the elastic energy and dissipation energy of the specimen begin to show a nonlinear growth trend. As the pore cracks are compacted and closed, the dissipation energy decreases, while the elastic energy rises. In the elastic deformation stage, the water-saturated specimen is pressurized to pressure, causing elastic deformation, which results in continuous increases in elastic energy and axial strain energy. During this stage, no cracks are produced, so the dissipation energy remains largely unchanged. As the specimen continues to deform, transitioning from the elastic stage to the plastic stage, some of the elastic deformation turns into plastic deformation. The growth rate of elastic energy decreases, while dissipation energy gradually increases due to the generation and expansion of new fissures and the expansion of natural defects in the water-saturated specimen. This leads to an increase in circumferential strain. In the plastic deformation stage, deformation continues until the destruction stage, where the bias stress decreases rapidly. This is accompanied by continuous increases in both axial and circumferential strains. The elastic energy is rapidly released, leading to intensified fissure expansion in the specimen, and the dissipation energy rises rapidly.

The environment of the confining pressure for the water-saturated material shows a significant pattern in the accumulation and dissipation of energy. In the compression stage, when the confining pressure increases from 0 MPa to 20 MPa, the total energy increases by 277%, with elastic energy increasing by 271% and dissipated energy increasing by 401%. This significant increase is due to the relatively small energy consumption during the compression stage, leading to a greater increase in energy. In the elastic stage, when the confining pressure increases from 0 to 20 MPa, the total energy increases by 237%, elastic energy increases by 236%, and dissipated energy remains unchanged, as there is minimal energy dissipation in the elastic stage. In the plastic stage, up to peak strength, when the confining pressure increases from 0 to 20 MPa, the total energy increases by 347%, elastic energy increases by 321%, and dissipated energy increases dramatically by 1028%. On the one hand, the existence of the surrounding pressure strengthens the density of the water-saturated material, thereby enhancing its energy storage capacity. On the other hand, the increase in surrounding pressure also leads to the extension of cracks in the water-saturated material, which requires the consumption of more energy.

### 5.3. Characterization of Energy Accumulation and Dissipation

**Figure 17:**
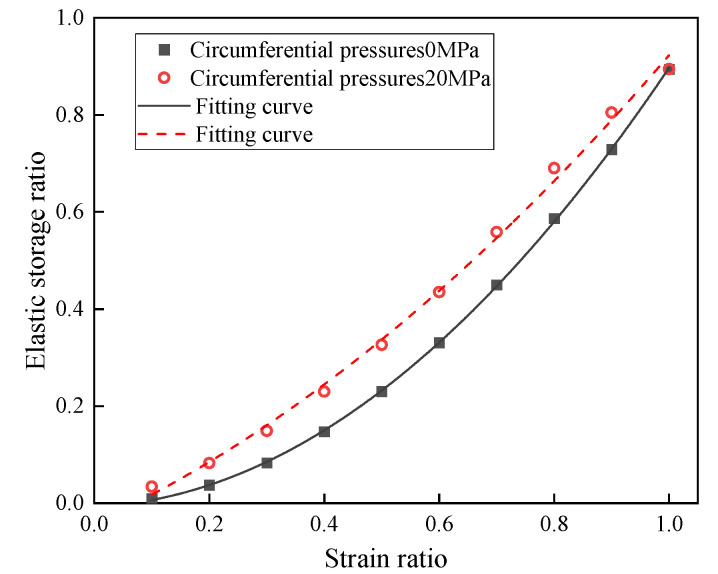
Variation curve of water-saturated defective material with strain ratio.

**Figure 18:**
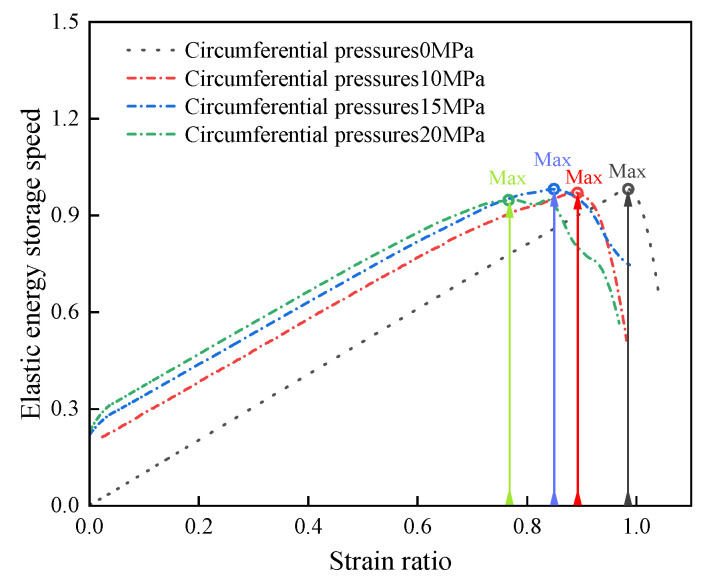
Characteristics of energy evolution rate change of water-saturated defective materials.

To quantitatively analyze the energy storage and dissipation characteristics of water-saturated defective rock materials, the elastic storage ratio is defined as the ratio of elastic energy to the maximum total energy, the dissipation ratio as the ratio of dissipated energy to the maximum total energy, and the displacement ratio as the ratio of the actual strain to the strain corresponding to the peak load. The variation in the elastic storage ratio with the strain ratio for water-saturated materials without confining pressure and with confining pressure of 20 MPa is illustrated in Figure 17, with the corresponding fitted functions provided in Equations (4) and (5). Therefore, the velocity of elastic storage ratio with strain ratio can be obtained as shown in Figure 18.

(4) $$y=0.85183x^{2}+0.05236x-0.00698$$

(5) $$y=0.41505x^{2}+0.54946-0.04138$$

This can be obtained through Figure 17 and Figure 18:

(1) The elastic energy storage ratios of defective rock materials with and without pressure enclosure and water saturation show nonlinear changes. Specifically, the elastic energy storage ratio increases with the strain ratio prior to reaching the peak load.
(2) The elastic energy storage ratios of materials with and without confining pressure are essentially similar during the compression stage. However, in the elastic and plastic phases, the elastic energy storage ratios of materials with confining pressure are higher than those without confining pressure. In the peak phase, the elastic energy storage ratios of both materials reach their maximum values and are comparable.
(3) The elastic storage ratio of water-saturated materials with confining pressure is larger than that of water-saturated materials without confining pressure at any stage. When the strain ratio is <0.94, the elastic storage rate of water-saturated materials with higher confining pressure is the largest. However, when the strain ratio is >0.94, the elastic storage ratio of water-saturated materials with higher confining pressure is progressively smaller than that of materials with lower confining pressure.

The above analysis demonstrates that the presence of confining pressure enhances the energy storage performance of water-saturated materials. At strain ratios close to 1, the enclosing pressure reduces the storage ratio rate. This is due to the fact that the presence of enclosing pressure increases the dissipated energy required for the destruction of water-saturated materials, which in turn leads to the timing of energy release when the material is destroyed.

## 6. Discussion

This study investigates the influence of confining pressure on the mechanical properties, failure modes, and energy evolution mechanisms of water-saturated brittle materials with natural defects through triaxial compression experiments and discrete element numerical simulations. The findings exhibit both connections to and extensions of previous research, with specific discussions as follows:

(1) Energy Accumulation and Release Under Confining Pressure

Experimental results reveal that confining pressure significantly alters the magnitude of energy accumulation and release in water-saturated defective materials. When the confining pressure increases from 0 to 20 MPa, the elastic energy, dissipated energy, and total energy increase by 321%, 1028%, and 347%, respectively. This phenomenon challenges the established understanding from Meng et al. [20], who reported reduced elastic energy storage under uniaxial compression in water-bearing conditions. The results highlight a unique energy evolution mechanism driven by the combined effects of confining pressure and water saturation.

(2) Nonlinear Relationship Between Elastic Energy Storage Ratio and Strain Ratio

The nonlinear relationship between the elastic energy storage ratio and strain ratio demonstrates that the energy storage rate decreases under high confining pressure when the strain ratio exceeds 0.94. This observation contrasts with the findings of Huang et al. [28], who observed rapid energy dissipation under high confining pressure. The delayed energy release during quasi-static loading, caused by confining pressure prolonging the plastic deformation stage, provides critical insights for early warning systems in deep engineering projects.

(3) Structural Stability and Limitations

Confining pressure enhances structural stability by improving the material’s energy storage capacity. However, attention must be paid to the critical threshold of abrupt energy changes under high confining pressure. The use of a single lithology in this experiment limits the generalizability of the conclusions. Subsequent studies should expand the research to diverse lithological systems to validate and broaden these findings.

This discussion underscores the dual role of confining pressure in stabilizing materials while necessitating caution in energy-critical engineering applications. Future work addressing multi-lithology systems and coupled field effects will further refine the theoretical framework for deep geomechanical engineering.

## 7. Conclusions

Through triaxial compression tests conducted on water-saturated natural defective rock materials, the following conclusions are drawn from the investigation into the influence mechanism of confining pressure on the mechanical properties, damage characteristics, and energy evolution of these materials:

(1) The peak stress and elastic modulus of defective rock materials under dry conditions are higher than those under water-saturated conditions. Under different confining pressures, the compressive strength and elastic modulus of water-saturated materials exhibit a positive correlation with the level of confining pressure, while the Poisson’s ratio shows a negative correlation with the level of confining pressure. Specifically, as the confining pressure is raised from 0 MPa to 20 MPa, the compressive strength increases by 126.8%, the elastic modulus increases by 91.9%, and the Poisson’s ratio decreases by 39%.
(2) The critical stresses of defective rock materials in dry conditions are higher than those in water-saturated conditions. In the gradual process of damage of water-saturated materials, the crack closure stress, crack initiation stress, and damage stress exhibit a positive correlation with the level of confining pressure. Specifically, as the confining pressure increases from 0 MPa to 20 MPa, the crack closure stress grows by 42.7%, the crack initiation stress grows by 98.7%, and the damage stress grows by 124.2%. Furthermore, the confining pressure slows down the crack initiation and damage processes of water-saturated materials.
(3) The damage mode of water-saturated material shifted from tensile damage mode to shear damage mode under different confining pressures. Additionally, the level of confining pressure exhibited a negative correlation with the crack inclination angle. The angle of rupture of the material decreased from 87° to 50.3° as the confining pressure was raised from 0 MPa to 20 MPa.
(4) Under varying pressures, cracks in water-saturated materials initiated from the vertical direction around to the middle tilt direction. The crack extension direction was concentrated in the interval of 45~135°. Furthermore, the formation of shear cracks exhibited a strong positive correlation with the level of confining pressure. Specifically, the ratio of shear cracks increased from 12.1% to 27.7% as the confining pressure rose from 0 MPa to 20 MPa.
(5) The energy characteristics of water-saturated materials exhibit a significant positive correlation with the level of confining pressure. When the confining pressure increases from 0 MPa to 20 MPa, the total energy increases by 277%, the elastic energy increases by 271%, and the dissipation energy increases by 401%. Furthermore, the elastic storage rate generally displays a positive correlation with the level of confining pressure. However, a negative correlation appeared when the strain ratio was greater than 0.94.

## Figures and Tables

**Table 1:** Triaxial test program.

Pressurization/MPa	Waterlogged State		Dry State	
	Test Number	Loading Rate MPa/s	Test Number	Loading Rate MPa/s
0	BS-0	0.01	GZ-0	0.01
10	BS-10		GZ-10	
15	BS-15		GZ-15	
20	BS-20		GZ-20	

**Table 2:** Simulated fine view parameters.

Type	*R_min_*/mm	*R_max_*/*R_min_*/(mm)	*E_c_*/GPa	*μ*	*σ_c_*/MPa	*c*/MPa	*Ø*	*k_s_*/*k_n_*
water-saturated defective brittle material	0.9	1.66	5.1	0.3	10.9	22.2	38	1.5

Note: *R_min_* is the minimum particle radius; *R_max_*/R_min_ is the particle radius ratio; E_c_ is the particle contact modulus; k_n_/k_s_ is the particle stiffness ratio; μ is the particle friction factor; Ø is the angle of internal friction; σ_c_ is the parallel bond tensile strength; and c is the strength of parallel bond cohesion.

## Data Availability

The data are not publicly available due to privacy or ethical restrictions.

## References

[1] Li W., Zhang Q., Wang X., Yu L., Li Z. (2024). Synergistic effect of particle size, carboxymethyl starch and Na_2_CO_3_ on rheological and filtration property of bentonite-based material. Case Stud. Constr. Mater..

[2] Xu R., Zhao Y., Dou B., Hou R., Li Z., Dong J. (2025). Experimental investigation of the effect of strain rate on the cracking behaviour and acoustic emission characteristics of red sandstone containing orthogonal cross flaws under quasistatic uniaxial compression. Eng. Fail. Anal..

[3] Li W., Yu L., Zhang T., Su H., Mi X., Fan D., Jin B. (2024). Quantitative analysis of grain size effect on tensile mechanical behavior of granite based on multi-level force chain networks. Comput. Part. Mech..

[4] Xu R., Gao L., Jin Y., Wang Y., Li Z., Hu S. (2025). Influence of strain measurement methods on crack initiation and crack damage thresholds of shale. Geomech. Geophys. Geo Energy Geo Resour..

[5] Xie H., Zhang R., Zhang Z., Gao M., Li C., He Z., Li C., Liu T. (2023). Reflections and explorations on deep earth science and deepearth engineering technology. J. China Coal Soc..

[6] Tao M., Wu X., Zhao R. (2024). Advancements and future prospects of mechanical characteristics of rock stress gradients. J. Cent. S. Univ. (Sci. Technol.).

[7] Han J., Hu S., Gao Z., Huang J., Cheng Y., Guo S., Yang L. (2024). A discrete element study of the fracture propagation of rock-like materials under directional sleeve fracturing. Theor. Appl. Fract. Mech..

[8] Guo S., Hu S., Huang J., Gao Z., Cheng Y., Han J., Yang L. (2023). Stability control technology for surrounding rocks in gob-side entry driving with small coal pillars under dynamic pressure. Energies.

[9] Colback P.S.B., Wild B.L. (1965). Influence of moisture content on the compress strength of rock. Proceedings of the 3rd Canadian Rock Mechanics Symposium.

[10] Li W., Yu L., Tan Y., Wu L., Qian J. (2024). Mechanical properties and impact behavior of frozen clay: Insights from static mechanical tests, fly-plate tests, and split-Hopkinson pressure bar analysis. Phys. Fluids.

[11] Song C., Feng G., Bai J., Cui J., Wang K., Shi X., Qian R. (2023). Progressive failure characteristics and water-induced deterioration mechanism of fissured sandstone under water–rock interaction. Theor. Appl. Fract. Mech..

[12] Chen X., Eichhubl P., Olson J.E. (2017). Effect of water on critical and subcritical fracture properties of Woodford shale. J. Geophys. Res. Solid Earth.

[13] Zhao Y., Tang J., Wang W., Cheng G., Luo S., Fu C. (2018). Study on failure behavior of fluid-solid coupling under conventional triaxial compression for Maokou limestone. J. Min. Saf. Eng..

[14] Luo S., Yan P., Lu W., Dong Z., Zhou C., Yang Z., Hu Y. (2023). Stability index of surrounding rock during deep rock excavation considering energy release speed. Appl. Sci..

[15] Vardar O., Zhang C., Canbulat I., Hebblewhite B. (2019). Numerical modelling of strength and energy release characteristics of pillar-scale coal mass. J. Rock Mech. Geotech. Eng..

[16] Zhang W., Feng J., Ma J., Shi J. (2022). The revealed mechanism of rock burst based on an Innovative calculation method of rock mass released energy. Int. J. Environ. Res. Public Health.

[17] Hu S., Zhang C., Ru W., Han J., Guo S., Zhou X., Yang L. (2023). Creep properties and energy evolution characteristics of weakly cemented rock under step loading. Int. J. Rock Mech. Min. Sci..

[18] Zhang J., Song Z., Wang S. (2021). Experimental investigation on permeability and energy evolution characteristics of deep sandstone along a three-stage loading path. Bull. Eng. Geol. Environ..

[19] Xie H., Gao F., Yang J. (2015). Research and exploration of rock mechanics in deep ground engineering. Chin. J. Rock Mech. Eng..

[20] Meng Z.P., Pan J.N., Liu L.L., Meng G.X., Zhao Z.H. (2009). Influence of moisture contents on mechanical properties of sedimentary rock and its bursting potential. Chin. J. Rock Mech. Eng..

[21] Lai X., Zhang S., Cui F., Wang Z., Xu H., Fang X. (2020). Energy release law during the damage evolution of water-bearing coal and rockand pick-up of AE signals of key pregnancy disasters. Chin. J. Rock Mech. Eng..

[22] Liu W., Yan E., Dai H., Du Y., Xiao W.B., Zhao S. (2020). Study on characteristic strength and energy evolution law of Badong formationmudstone under water effect. Chin. J. Rock Mech. Eng..

[23] Liu Y., He C., Fu H., Wang S., Lei Y., Peng Y. (2020). Study on tensile mechanical properties and energy consumption law of saturatedslate under impact loads. Chin. J. Rock Mech. Eng..

[24] Guo J., Liu X., Qiao C. (2014). Experimental study of mechanical properties and energy mechanism of karst limestone under natural and saturated states. Chin. J. Rock Mech. Eng..

[25] Gao F., Cao S., Zhou K., Lin Y., Zhu L. (2020). Damage characteristics and energy-dissipation mechanism of frozen–thawed sandstone subjected to loading. Cold Reg. Sci. Technol..

[26] Yang Y., Ma D. (2018). Experimental research on energy evolution properties of coal sample failure under triaxial unloading testing. J. Min. Saf. Eng..

[27] Zhao Y., Gong S., Huang Y. (2015). Experimental study on energy dissipation characteristics of coal samples under impact loading. J. China Coal Soc..

[28] Huang M., Zhang J., Hu L., Zhang X. (2015). SplitHopkinson pressure bar test and energy dissipationcharacteristics of argillaceous siltstone in Three Gorgesreservoir region. J. Eng. Geol..

[29] Peng Y., Yu L., Qian J., Li W., Zhang T., Zhou L. (2025). Dynamic tensile behavior and crack propagation in coral aggregate seawater shotcrete: Experimental investigation and numerical simulation. Cem. Concr. Compos..

[30] Jiang J., Chen S., Xu J., Liu Q. (2018). Mechanical properties and energy characteristics of mudstone under different containing moisture states. J. China Coal Soc..

[31] Ru W., Hu S., Li D., Ma J., Zhang C., Luo P., Gong H., Zhou A. (2023). Energy evolution of unloading confining pressure and dissipative energy damage constitutive model of coal-rock combination. J. Rock Soil Mech..

